# Reproducibility of improvements in patient-reported functional ability following functional capacity evaluation

**DOI:** 10.1186/s12891-022-05208-w

**Published:** 2022-03-16

**Authors:** Martin Schindl, Harald Zipko, Matthias Bethge

**Affiliations:** 1 Rehab Center Weißer Hof, AUVA, Holzgasse 350, Klosterneuburg , A-3400 Austria; 2 FH Campus Wien, Favoritenstrasse 226, Wien, 1100 Austria; 3 Institute of Epidemiology and Social Medicine, University of Lübeck, Ratzeburger Allee 160, Lübeck, 23562 Germany

**Keywords:** Trauma, Rehabilitation, Return to work, Functional capacity evaluation, Cohort study, Replication, Reproducibility, Diagnostic

## Abstract

**Background:**

Performance of functional capacity evaluation (FCE) may affect patients, self-efficacy to complete physical activity tasks. First evidence from a diagnostic before-after study indicates a significant increase of patient-reported functional ability. Our study set out to test the reproducibility of these results.

**Methods:**

Patients with musculoskeletal trauma and an unclear return to work prognosis were recruited in a trauma rehabilitation center in Lower Austria. We included patient cohorts of three consecutive years (2016: *n* = 161, 2017: *n* = 140; 2018: *n* = 151). Our primary outcome was patient-reported functional ability, measured using the Spinal Function Sort (SFS). SFS scores were assessed before and after performing an FCE to describe the change in patient-reported functional ability (cohort study). We investigated whether the change in SFS scores observed after performing an FCE in our first cohort could be replicated in subsequent cohorts.

**Results:**

Demographic data (gender, age and time after trauma) did not differ significantly between the three patient cohorts. Correlation analysis showed highly associated before and after SFS scores in each cohort (2016: r_s_ = 0.84, 95% CI: 0.79 to 0.89; 2017: r_s_ = 0.85, 95% CI: 0.81 to 0.91; 2018: r_s_ = 0.86, 95% CI: 0.82 to 0.91). Improvements in SFS scores were consistent across the cohorts, with overlapping 95% confidence intervals (2016: 14.8, 95% CI: 11.3 to 18.2; 2017: 14.8, 95% CI: 11.5 to 18.0; 2018: 15.2, 95% CI: 12.0 to 18.4). Similarity in SFS scores and SFS differences were also supported by non-significant Kruskal–Wallis H tests (before FCE: *p* = 0.517; after FCE: *p* = 0.531; SFS differences: *p* = 0.931).

**Conclusions:**

A significant increase in patient-reported functional ability after FCE was found in the original study and the results could be reproduced in two subsequent cohorts.

**Supplementary Information:**

The online version contains supplementary material available at 10.1186/s12891-022-05208-w.

## Background

Reproducibility is a core principle of scientific progress [1, 2]. In 2015, a much-noticed paper by a collective of researchers showed that a great number of research findings in the field of psychology could not be replicated. While 97% of the original studies reported significant results, only 36% of the replication studies agreed. Furthermore, the reported effect sizes were only about half as large as those in the original publications [3]. Not surprisingly, about half of the researchers who responded to a Nature survey about reproducibility recognized a significant crisis of reproducibility [4].

Rehabilitation researchers have made similar observations. Maytas and Ottenbacher highlighted the high number of non-reproducible findings in stroke rehabilitation research almost 30 years ago, and demonstrated that unfounded hypotheses and low power almost inevitably lead to false alarms and random findings [5]. This finally results in a state of research characterized by contradictory results. Since then, the call for replication studies has grown louder in many scientific fields [6, 7].

In rehabilitation and occupational medicine, functional capacity evaluation (FCE) is used to assess functional capacity and to guide occupational rehabilitation programs [8–11]. This includes patients with low back pain [12], whiplash injury [13], musculoskeletal mono- and polytrauma [14], as well as amputations of the upper extremity [15]. In a recent study following patients who had experienced trauma, it was indicated that FCE, when used as a diagnostic procedure, may also have a direct therapeutic effect on a patient’s self-reported functional ability [14]. Using the Spinal Function Sort (SFS), a picture-based patient-reported measure of functional ability, prior to and directly after the FCE, a significant increase was found after completion of an FCE. It was reasoned that this effect might have been driven by allowing a realistic appraisal of the ability to perform relevant work activities, and thus, contribute to a higher patient experienced self-efficacy. The clinical significance of this finding is supported by recent studies showing that patient-reported functional ability is a prognostic measure of return-to-work [16]. Since the direct therapeutic effect of a diagnostic FCE reported in the original study represents some novelty, we aimed to reproduce these findings. To do this, analyses were repeated in two subsequent patient cohorts in 2017 and 2018, treated in the same rehabilitation setting. In these subsequent studies, we aimed to repeat the experimental and contextual conditions of the primary study as closely as possible, to control sampling error and chance, and give the best chance of direct reproducibility [17]. Our main aim was to compare the improvements in self-reported functional ability in the original cohort and two subsequent patient cohorts recruited in same rehabilitation center.

## Methods

### Study design

We assessed the reproducibility of the findings of our initial cohort study (recruited in 2016) by following two subsequent patient cohorts (recruited in 2017 and 2018) that completed the same diagnostic before–after study as the initial cohort [14]. We did not use a control group because our original study also applied a before-after design. In brief, patients rated their functional ability before and after FCE. The FCE was performed on two consecutive days. The performance on two days is the standard procedure and is intended to avoid overloading patients. Assessing self-reported functional ability twice before and immediately after performing an FCE is also part of the standardized FCE protocol. The time between pretest and posttest assessment was short to minimize the risk of interfering factors influencing patient perception of their functional ability, e.g. improvement due to the natural course of musculoskeletal trauma-related disorders or effects of an intervention. For consistency, the same rehabilitation unit, testing therapists, medical staff, FCE protocol and primary outcome were kept the same while repeating the study in two different populations. The two subsequent studies were performed on cohorts of patients who had experienced trauma and had been referred to FCE due to uncertainty in the possibility of returning to work. The original sample size was determined by the inclusion of all eligible patients, and we assumed that comparable sized samples could be recruited in the following two years. The original study and the recruitment of the following two cohorts were reviewed and approved by Ethics Committee of the Provincial Government of Lower Austria (GS1-EK-4/502–2017). We used the STROBE checklist when preparing the manuscript to ensure transparent and complete reporting of our study design and findings [18].

### Setting

All patients were treated in the same 200-bed in-patient trauma rehabilitation center in Lower Austria, Austria. In 2016, approximately 1200 patients were treated in our unit. (For further details refer to https://www.auva.at/cdscontent/?contentid=10007.670948).

### Participants

In the original study [14], patients were assigned to our inpatient rehabilitation center located in the eastern part of Austria following a non-work or work accident (monotrauma or polytrauma, burns, amputation, and spinal cord injury). In 2016, approximately 1200 patients were treated in our unit. While the rehabilitation program is a multi-professional one, work-related functional capacity training and other work-related treatment components are not routinely used in these programs. In 2016 patients were referred to FCE if, at the end of the inpatient rehabilitation program, the rehabilitation team was uncertain as to whether the patient was able to perform the work demands of their previous job. If the team considered a return to work was likely, patients were not referred for FCE. Patients who were referred for FCE were eligible for the study. A rehabilitation physician checked the inclusion and exclusion criteria including medical stability status, ambulation without walking aids, and the ability to read and understand German. Patients in the following years were eligible for study inclusion, if they fulfilled the same inclusion and exclusion criteria as in the year 2016.

### Intervention

The WorkWell Systems FCE was developed by Susan Isernhagen in the 1990s as a systematic method to observe a subject’s ability to perform work-related tasks [8, 19, 20].The complete test battery consists of 29 items in 5 performance categories (weight handling and strength, posture and mobility, locomotion, balance, and hand coordination). For the 6 weight handling tests, the tasks must be repeatedly performed while the load is gradually increased to the level of maximal safe performance. Other tests use norms (for example, grip strength, walking speed, hand co-ordination), while in posture and mobility tests time ceiling (for example, working overhead that is performed for 5 min) or qualitative descriptors such as movement patterns, base of support, posture, and order of muscle recruitment are used to describe the respective functional capacity (for example pushing a weighted cart over a distance of 9 m) to terminate a test.

The FCE was performed on two consecutive half-days, with a therapy-free afternoon between the two test days.

The FCE was administered by either a physiotherapist or occupational therapist experienced in the FCE procedure. All therapists had done at least 10 FCEs per year during the least 5 years. The final report was then confirmed by one of two rehabilitation physicians with 5–10 years of experience in performing FCE. Both physicians had performed approximately 50 to 80 FCEs per year during the least 5 years. All therapists and rehabilitation physicians were trained and certified to perform the WorkWell Systems FCE.

### Measures

#### Self-reported functional ability

Our primary outcome was the Spinal Function Sort (SFS). The SFS is a picture-based questionnaire, including 50 items that assess the patient’s ability to perform various work tasks and instrumental activities of daily living (for example, picking up a small tool, lifting a 10 kg tool box, or climbing a ladder) [16, 21, 22]. The SFS was originated in the 1980s to assess self-reported function capacity in low back pain patients. However, the SFS has been reported to be valid and reliable also in patients with other musculoskeletal problems including whiplash injuries and trauma [22, 23]. Moreover, the SFS was shown to be a predictor of return-to-work [16]. Items are 5-point scaled from “unable” to “able”. A total score was calculated ranging from 0–200 points, with higher scores indicating better perceived functional capacity. The SFS was completed by the patient before testing and a second time after the FCE was finalized on the second day. This immediate assessment was used to reduce the effect of other treatments.

#### Other variables

We additionally assessed age, sex and time between injury and start of the rehabilitation program to describe the samples. All data generated or analyzed during this study are included in this published article and its supplementary information files.

### Statistics

Patient cohorts were described with absolute frequencies, means, medians and 95% confidence intervals (CIs) plus corresponding graphs (box and whisker plots, density plots, scatterplots).

Our approach of statistical analysis in the present replication study did not aim to gain non-significant p-values. We mainly compared the figures of the first cohort and their associated 95% confidence intervals, with estimates for the two subsequent cohorts. In more detail, reproducibility of findings of the first cohort in the two succeeding cohorts was analyzed in a number of ways. For rank correlation analysis based on scatterplots including straight linear regression lines we calculated Spearman’s r_s_. Reproducibility was achieved when Spearman’s r_s_ of every cohort was included in the 95% CIs of the other cohorts as described by Zou [24]. For comparison of regression estimates we calculated regression slopes and considered reproducibility if the slope of every cohort was included in the 95% CIs of the other cohorts. For graphic analysis of reproducibility, we used Bland–Altman scatterplots with corresponding data ellipse density plots. Reproducibility was determined when confidence intervals for 95% limits of agreement of straight regression lines overlapped.

Although we primarily inspected the overlap of the confidence intervals, we additionally calculated hypothesis tests to present further evidence of reproducibility. We used Fisher’s exact test for categorical variables and the Kruskal–Wallis H test for continuous variables. We also compared the slopes for regressing posttest SFS scores on pretest SFS scores using an analysis of variance with Tukey’s p-value adjustment. For hypothesis tests the type I error was set to 20%, and reproducibility was achieved when the probability of error exceeded 20%.

Statistical and graphical analyses were performed using the basic version of R 3.6.1 with dedicated standard packages (car, bestNormalize, boot, cocor, psych and lsmeans). We have provided our data as Supplementary File 1.

## Results

### Sample and demographic variables

161 patients were included in the first cohort recruited in 2016, followed by 140 subjects in 2017 and finally 151 participants in 2018 (Table 1). Overall demographic and clinical data did not differ significantly between the three patient cohorts. About half of the patients had one affected body part. About three quarter had to cope with a heavy or very heavy work load. Table 2 summarizes the results of reproducibility analysis.

**Table 1:** Sociodemographic data

	**2016**	**2017**	**2018**	
	*n* = *161*	*n* = *140*	*n* = *151*	
	% or mean	% or mean	% or mean	*p*
Sex				0.584^a^
Male	89	92	89	
Age (years)	42.7 [41.0; 44.4]	43.4 [41.6; 45.3]	44.6 [42.9; 46.3]	0.351^b^
Time after trauma (months)	13.4 [10.0; 16.8]	11.8 [9.0; 14.6]	13.5 [8.8; 18.2]	0.607^b^
Marital status				0.856^a^
Married/partnership	68	69	72	
Single/divorced/parted	32	31	28	
Minor children (Yes)	45	46	47	0.989^a^
Native language				0.979^a^
German	73	69	68	
Serbian/Croatian/Bosnian	8	12	12	
Turkish	4	3	5	
Polish	4	3	2	
Hungarian	2	4	3	
Slovakian	1	3	1	
Macedonian	1	1	1	
Kosovan	1	1	3	
Greek	0	0	1	
Italian	0	0	1	
Others	6	4	5	
Work contract (Yes)	52	50	62	0.856^a^
Work load (DOT category)				0.203^a^
Sedentary	0	1	0	
Light	1	1	1	
Medium	22	21	23	
Heavy	39	38	36	
Very heavy	32	39	40	
Unknown	6	0	0	
Number of affected body parts (i.e. musculoskeletal regions: arm, hand, trunk, leg, foot, % of patients)				0.154^a^
1	57	42	58	
2	34	41	32	
≥ 3	9	17	10	

Age and time after trauma are presented as mean with 95% confidence intervals, ^a^Fisher’s exact test, ^b^Kruskal-Wallis H tests, *DOT* Dictionary of Occupational Titles [25]

**Table 2:** Summarized results of reproducibility analysis

	2016	2017	2018	*p*
	*n* = 161	*n* = 140	*n* = 151	
SFS before FCE	135.5 [129.3; 141.7]	136.3 [129.8; 142.8]	132.8 [126.9; 138.8]	0.517^a^
SFS after FCE	150.3 [144.7; 156.0]	151.1 [145.8; 156.4]	148.1 [142.9; 153.2]	0.531^a^
Spearman’s r	0.836 [0.783; 0.896]	0.855 [0.809; 0.908]	0.861 [0.820; 0.909]	
2016 vs. 2017				0.578^b^
2016 vs. 2018				0.444^b^
2017 vs. 2018				0.849^b^
SFS difference	14.8 [11.3; 18.2]	14.8 [11.5; 18.0]	15.3 [12.0; 18.4]	0.934^a^

SFS scores and improvement of the SFS score from before measurement are presented as mean with 95% confidence intervals. Spearman’s r describes the correlation of SFS scores before and after performance of the FCE and is presented with 95% confidence intervals based on bootstrap analysis based on 10.000 replicas. Superscripts indicate hypothesis tests with α = 20% to support similar results. ^a^Kruskal-Wallis H tests, ^b^pairwise tests for correlations, *SFS* Spinal Function Sort, *FCE* functional capacity evaluation

### Functional ability before and after FCE

**Fig. 1:**
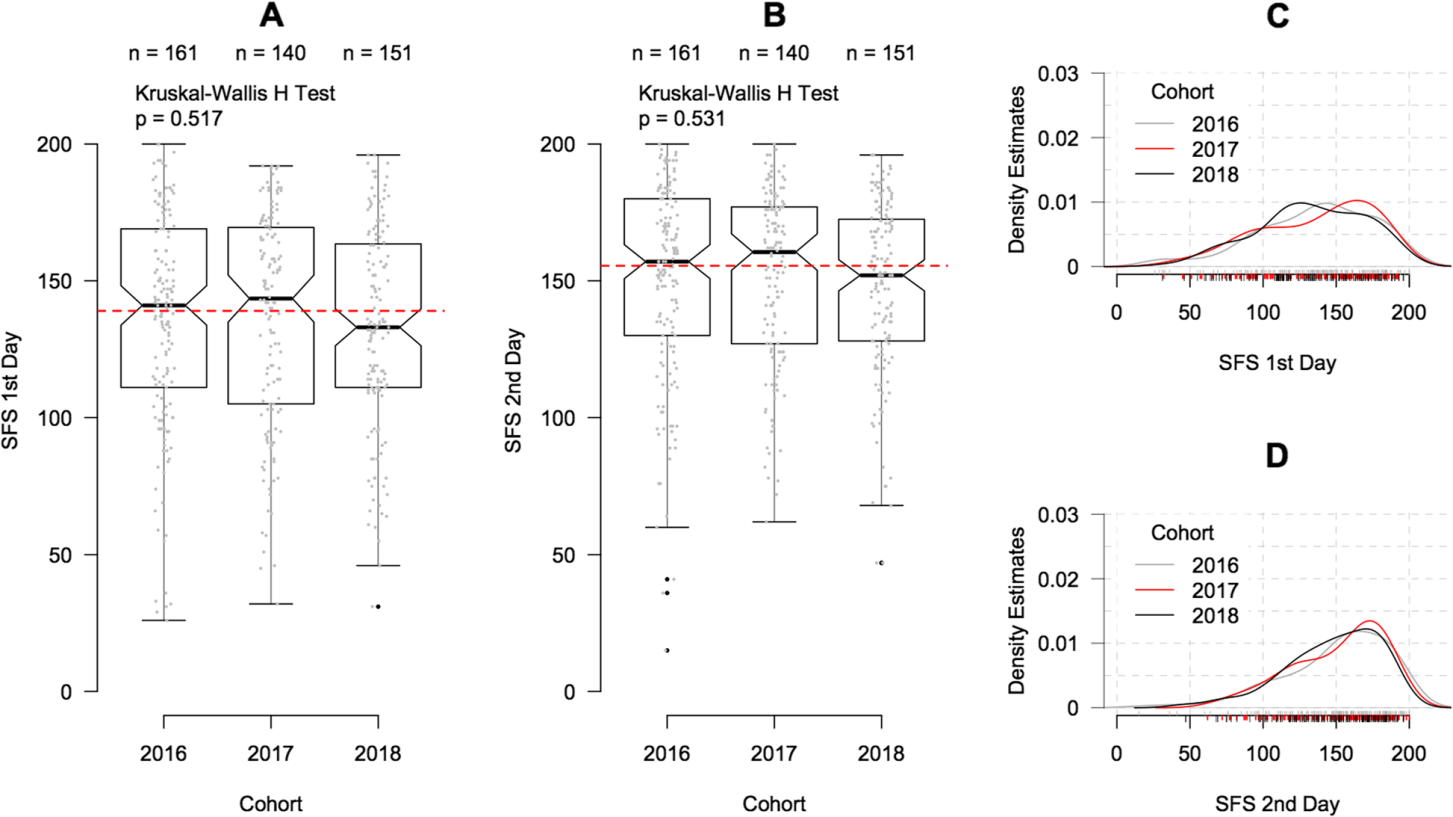
Distribution of SFS scores. **A** and **B** box and whisker plots of first and second day SFS scores (overall median reflected by red-dotted lines) **C** and **D** density plots for first and second day SFS scores. SFS: Spinal Function Sort

The distributions of the SFS scores are presented in Fig. 1. The distributions were very similar across the three cohorts and were slightly left-skewed, with the mean to the left of the peak. As expected, distributions of the SFS scores in the three cohorts highly overlapped, and neither the SFS scores before the FCE nor the SFS scores after the FCE differed significantly between the three cohorts.

**Fig. 2:**
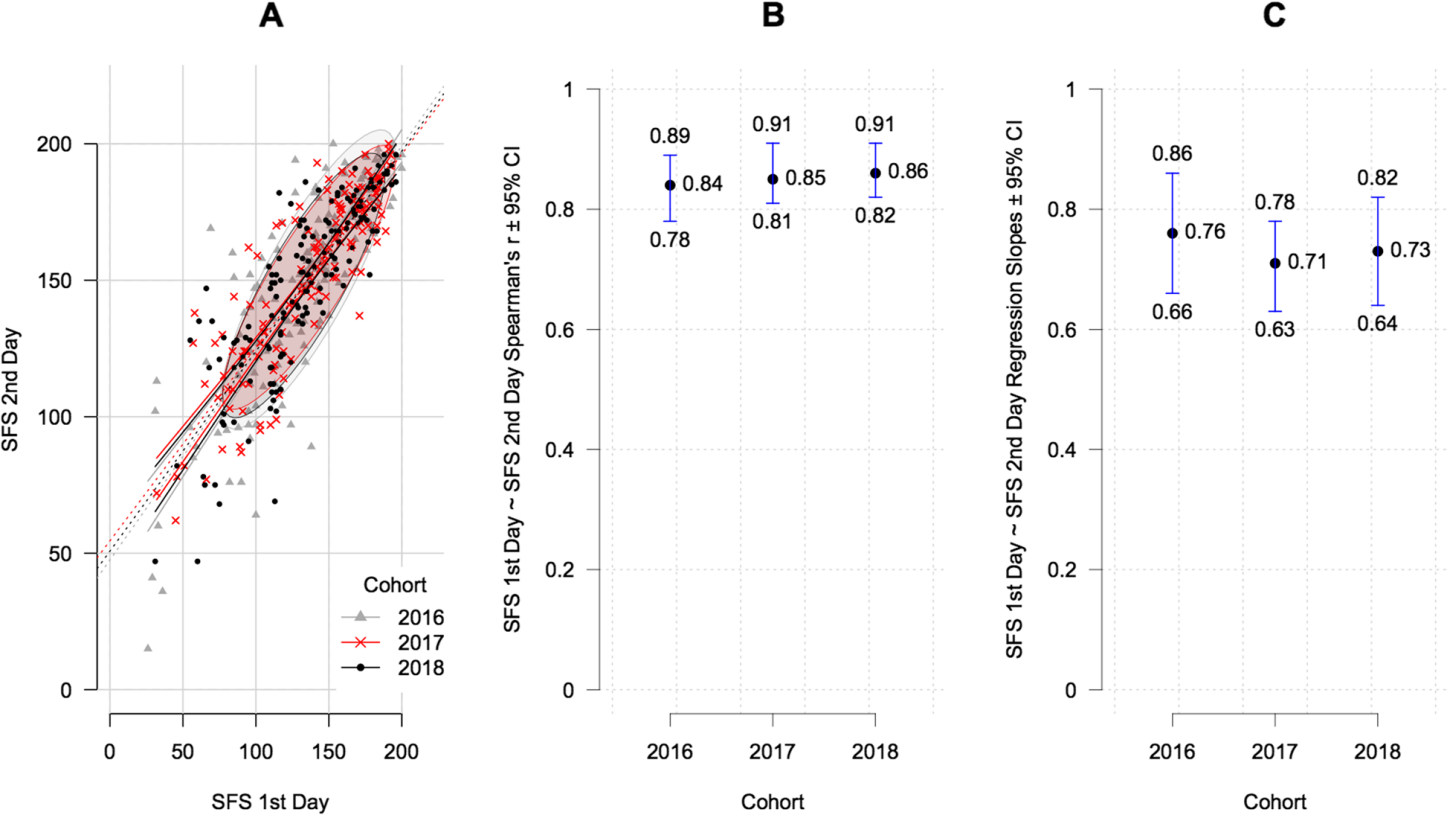
Correlation of SFS scores before and after performing the FCE. **A** scatterplots with data density ellipses representing two thirds of patient data and dotted lines indicating linear regression lines with the corresponding 95% CI (solid lines); **B** and **C**: Spearman’s correlation and regression coefficients with associated 95% CI. SFS: Spinal Function Sort; FCE: functional capacity evaluation; CI: confidence interval

Both bivariate scatterplots, and Spearman’s rank correlation analysis calculated with each patient’s SFS scores from the first and second day, revealed notable correlation estimates with r_s_ > 0.8 (Table 1, Fig. 2 A-C). CIs of the correlation estimates highly overlapped. Pairwise comparison of Spearman’s r_s_ did not show any significant differences between the cohorts (Table 1, Fig. 2B; 2016 vs. 2017: 95% CI: -0.085 to 0.048, *p* = 0.578; 2016 vs. 2018: 95% CI: -0.089 to 0.039, *p* = 0.444; 2017 vs. 2018: 95% CI: -0.070 to 0.056, *p* = 0.849). Neither the comparison of the linear regression slope estimates and their 95% CIs, nor the ANOVA based pairwise comparisons of the slope estimates, revealed significant differences (2016 vs. 2017: *p* = 0.611; 2016 vs. 2018: *p* = 0.879; 2017 vs. 2018: *p* = 0.902).

### Improvement in functional ability

**Fig. 3:**
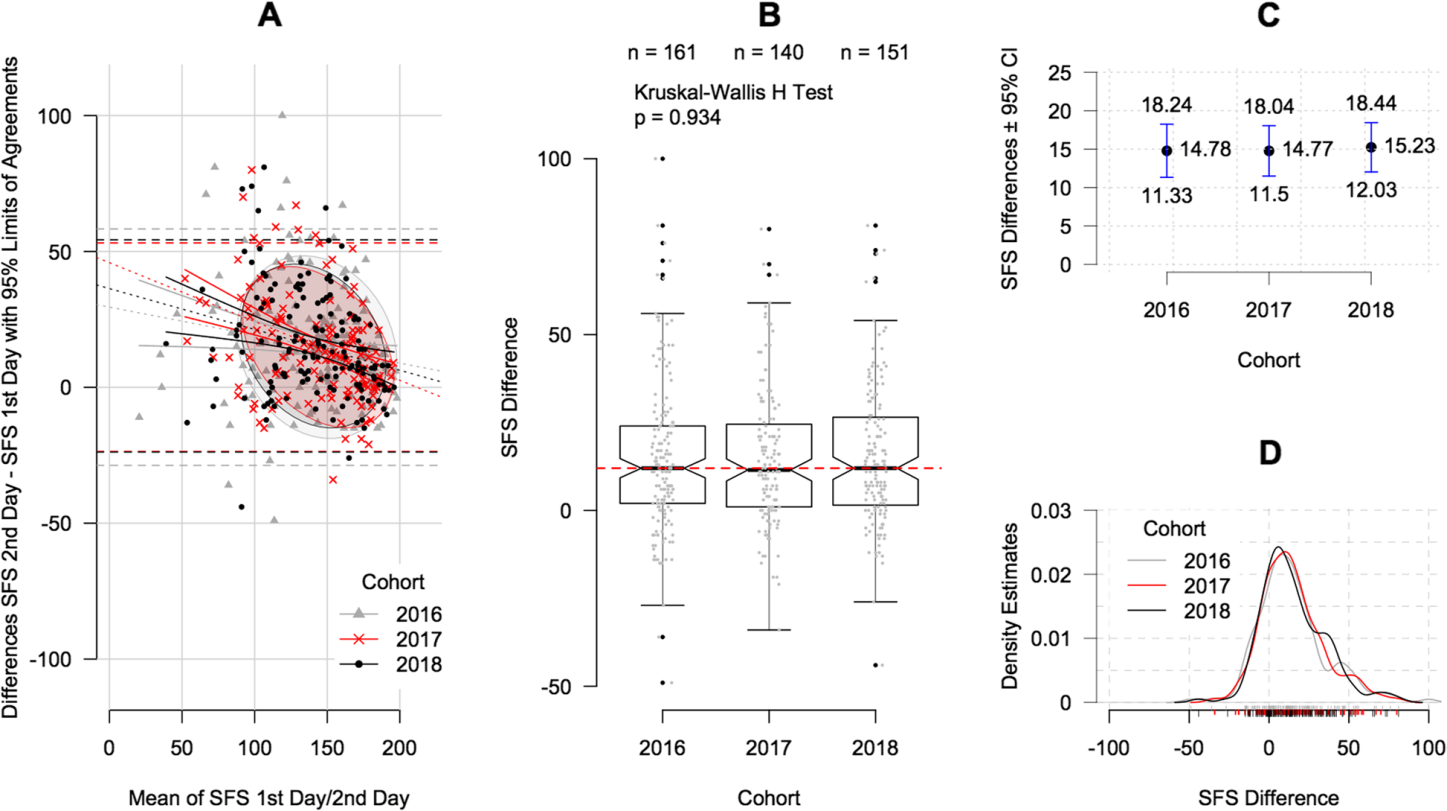
Improvement in functional ability. **A** Bland–Altman plots (ellipses representing two thirds of patient data, dotted lines indicating linear regression lines with solid lines representing corresponding 95% CI); **B** Box and whisker plots of SFS score differences (overall median reflected by red-dotted lines); **C** mean SFS differences in each cohort with related 95% CI; **D** density plots showing the overlapping positive increase in SFS scores

Graphical analyses using Bland–Altman plots showed that most patients had higher SFS scores after the FCE (Fig. 3A). A higher mean SFS score before and after FCE measurement was associated with less improvement. Both data ellipse densities, and 95% CIs of the regression line depicted in the Bland–Altman plots showed highly corresponding estimate areas. The analysis of SFS score changes after the FCE showed no evidence of significant differences between the three cohorts (*p* = 0.934; Table 1, Fig. 3B). In addition, in 2016, 2017 and 2018, patient-reported functional ability (0–200 points) improved by 14.8 (95% CI: 11.3 to 18.2), 14.8 (95% CI: 11.5 to 18.0) and 15.2 points (95% CI: 12.0 to 18.4), respectively (Fig. 3C). The comparison of each patient’s SFS score difference revealed a remarkably stable distribution, with an estimated average gain of 14.9 points (95% CI: 13.0 to 16.8) (Fig. 3D).

## Discussion

Reproducibility is a cornerstone of research [1]. In this study we aimed to report on the direct reproducibility of the increase in self-reported functional ability after performing FCE in patients with trauma. In the original study a statistically significant increase in patient-reported functional ability was reported after exposure to the WorkWell Systems FCE [14]. We suggested that the increase on the SFS reflects that the performance of an FCE positively influences the patient’s perception of his or her actual functional ability. This represented some novelty since the FCE protocol is mainly used as a diagnostic functional assessment and not as a therapeutic tool to modify patient perception. Therefore, we intended to directly reproduce our findings in two complete patient cohorts who were referred for FCE in the subsequent years. This approach was chosen to control for sampling error and chance. Our findings reproduced the statistically significant improvement in patient-reported functional ability after performing the two-day WorkWell Systems FCE, whilst also revealing a remarkably stable quantitative improvement across all cohorts.

As stated in Greenland et al. [26] our analysis of SFS scores was focused mainly on estimates and corresponding CIs to avoid any misinterpretation of p-values based on simple hypothesis testing. Reproducibility of the direction of the change (increased SFS scores after FCE) and the amount of the change was initially demonstrated in graphical analyses, and our comparison of CIs revealed comparable estimates in the three patient cohorts. Hypothesis tests (Kruskal–Wallis H test or Fisher’s exact test) were calculated to support the assumptions of the overall similarity of replicated findings, since p-values are not capable of measuring effect sizes or remarkable associations [27]. Similar estimates and corresponding 95% CIs confirmed that the absolute gain of SFS scores was within a stable probability range of our initial results [14].

Although an increase of approximately 15 points on the SFS is only an 8% score increase, this can equate to a significant increase in a patient’s strength and ability to perform work-related tasks (for example, being able to lift 5–10 kg more). We regard this a clinically important improvement [28]. It also important to note that the increase in SFS score of 15 points is an average, and, therefore, also includes the outcomes of so-called inconsistent patients who rated their personal work capacity lower than the observer during the FCE procedure. Had these patients been excluded, an even greater increase in the SFS score would have been noticed. The increase of 15 points after two days exposure to the quasi-realistic work environment of the FCE is approximately half that seen when patients rated their functional capacity after completion of a 4-week in-patient rehabilitation period [28].

Our findings are in line with Büschel and colleagues who considered an increase of at least 11 points on the SFS as clinically relevant [29]. Not only did these authors apply the SFS before and after the FCE procedure, but they also interviewed patients to determine if the FCE procedure had changed their perception of their functional capacity. In their study, 39.7% of the patients were surprised by, and pleased with, the increase in functional capacity that they were experiencing. However, 34.2% thought that the FCE did not change their perception and 24.7% overestimated their functional capacity prior to the FCE procedure. This direct report of a change in perception of functional ability was also reflected by an increase in the SFS score. An increase of at least 11 points was seen in 43.8% of patients, while only 16.4% showed a decrease of at least 11 points. The authors classified 39.7% of patients as unchanged. Moreover, Bühne and colleagues also reported increases in SFS scores of about 11 points, using an alternative FCE (not the WorkWell Systems FCE) [30]. This latter finding is noteworthy because it reproduces a similar change in functional ability when performing a different FCE, and can be interpreted as conceptual replication.

### Study limitations

Firstly, we did not aim to report on conceptual replication as this would have required alternative experimental or methodological approaches to gain additional evidence. This could be addressed by testing the underlying hypothesis that a patient has a better awareness of their functional ability and self-efficacy by performing the test. An alternative patient-reported measure that directly assesses self-efficacy or an alternative FCE protocol could be used for this, ideally in a randomized controlled trial. Secondly, a learning or practice effect is possible when repeatedly completing a questionnaire, and the improvement which we observed may at least partly be due to the short interval of completing the questionnaire again [31]. Matheson and Matheson reported high correlations between test and retest SFS scores in several test–retest reliability studies, but also indicated that an improvement is likely when studying test–retest reliability within rehabilitation settings [21]. However, in recent studies of the test–retest reliability of the French and German versions of the SFS, the mean change was as low as 0.3 and 1.3 points, respectively, when the SFS was completed on two occasions, separated by two days [23]. In addition, Trippolini and colleagues reported a change of only 0.2 points in a test–retest study of a sample of patients with sub-acute whiplash-associated disorders that were tested twice within a week [22]. We assume that mere recall of first-time responses is not very likely due to the high number of 50 questions when the SFS is reprocessed after 48 h. Therefore, we are confident that the change is not primarily due to the short time interval between completing the two questionnaires. Thirdly, another weakness is the consistently small number of female participants across all cohorts. Fourthly, we provide no evidence that the improvement we observed is lasting. We assume that a lasting effect needs repeated training of work functions. Many rehabilitation programs aiming to return patients to work rely on practicing work activities, similar to those tested during FCE, and there is increasing evidence to show that these programs successfully improve return to work [11]. Lastly, since we aimed to report on direct reproducibility, the study was performed in the same rehabilitation unit and by the same researchers as the initial cohort study. Therefore, a generalization of our results should be considered very cautiously. We provide evidence just for temporal reproducibility in very similar patient cohorts and recommend that the study is repeated in different rehabilitation units and patient groups, to further address the aspect of generalization.

### Study strengths

Firstly, data were collected as part of the clinical routine, regardless of the trial, therefore a Hawthorne effect due to patient’s knowledge of participating in a study is unlikely. Secondly, we used a patient-reported outcome measure, which is regarded to be more reflective of the real life of the patient [32]. The main outcome variable used in the study, the SFS, has been reported to have excellent test–retest reliability and construct validity [21–23, 33]. Moreover, the SFS has been used previously to predict return to work in patients with different medical conditions of the musculoskeletal system [16, 22]. Thirdly, we not only replicated our results once, but in two subsequent patient cohorts. In total, our findings are based on approximately 450 patients. Lastly, we provide free access to our data and have provided the data as a supplementary file to our manuscript.

## Conclusions

Overall, a significant increase in patient-reported functional ability after FCE was found in patients with musculoskeletal trauma in the original study, and the results were reproduced in two subsequent cohorts. We conclude that completion of the two-day WorkWell Systems FCE improves a patient’s self-reported functional ability. Our comparisons of robust estimations, corresponding 95% confidence intervals and graphical analyses across the three cohorts showed good reproducibility of results.

## Supplementary Information

**Additional file 1.**

## Data Availability

Data are enclosed.

## Abbreviations

ANOVA: Analysis of variance
CI: Confidence interval
FCE: Functional capacity evaluation
r_s_: Spearman’s rank correlation coefficient
SFS: Spinal Function Sort
STROBE: Strengthening the reporting of observational studies in epidemiology
